# The role of scene summary statistics in object recognition

**DOI:** 10.1038/s41598-018-32991-1

**Published:** 2018-10-02

**Authors:** Tim Lauer, Tim H. W. Cornelissen, Dejan Draschkow, Verena Willenbockel, Melissa L.-H. Võ

**Affiliations:** 1 Scene Grammar Lab, Department of Psychology, Goethe University Frankfurt, Frankfurt am Main, Germany; 2 Department of Psychology, University of Victoria, Victoria, BC Canada

## Abstract

Objects that are semantically related to the visual scene context are typically better recognized than unrelated objects. While context effects on object recognition are well studied, the question which particular visual information of an object’s surroundings modulates its semantic processing is still unresolved. Typically, one would expect contextual influences to arise from high-level, semantic components of a scene but what if even low-level features could modulate object processing? Here, we generated seemingly meaningless textures of real-world scenes, which preserved similar summary statistics but discarded spatial layout information. In Experiment 1, participants categorized such textures better than colour controls that lacked higher-order scene statistics while original scenes resulted in the highest performance. In Experiment 2, participants recognized briefly presented consistent objects on scenes significantly better than inconsistent objects, whereas on textures, consistent objects were recognized only slightly more accurately. In Experiment 3, we recorded event-related potentials and observed a pronounced mid-central negativity in the N300/N400 time windows for inconsistent relative to consistent objects on scenes. Critically, inconsistent objects on textures also triggered N300/N400 effects with a comparable time course, though less pronounced. Our results suggest that a scene’s low-level features contribute to the effective processing of objects in complex real-world environments.

## Introduction

Objects typically do not appear randomly in their surroundings. The regularity of object-scene co-occurrences helps us to give meaning quickly to our visual world, for instance, by predicting that the item on the kitchen counter is a mug and not a roll of toilet paper. Using line drawings, it was first demonstrated that objects that are semantically related to their scene context are detected faster and more accurately than objects that are unpredicted by the context^1^. But even very briefly presented naturalistic scenes modulate object processing such that objects placed on task-irrelevant background images are named more accurately if they are related compared to unrelated with the background scenes^2^ — which we refer to as semantic consistency effect. Importantly, this observation does not stem from basic feature overlap (e.g., shared shape or colour) and is replicable with focused attention directed to the object and the background, respectively^3^. In addition to behavioural measures that show effects of object-scene inconsistencies — like impaired object recognition performance — event-related potentials (ERPs) provide a sensitive measure that has been shown to track semantic processing via the N400 response, without the need for explicit responses by the observers. The N400 is an ERP component that has first been associated with semantic access to language^4,5^ but also to objects and scenes^6–9^ (for a review see^10^): Like reading an inconsistent word in a sentence, seeing an object that semantically violates its scene context elicits a negativity peaking about 400 ms post stimulus onset and typically evolving over the mid-central scalp region. Typically, semantically inconsistent objects in scenes also trigger an earlier effect known as the N300^7–9^ that may reflect rather pre-semantic, perceptual effects of context on object processing. However, the question whether the N300 and N400 ultimately reflect distinct underlying processes is still debated^11,12^.

What information of a scene is sufficient to modulate the semantic processing of objects in their visual context and actually drives the consistency effect? It is conceivable that scene context influences object processing in two different ways: One way to boost object recognition would be to use existing knowledge of frequent object co-occurrences (e.g., the item in question below the screen is probably a keyboard and not a cutting board). However, for this to work, objects within the scene would need to be identified first (the screen would need to be identified before it can influence identification of the object below it), which could be too time consuming for the context to exhibit rapid consistency effects. Therefore, more global properties of a scene might also play a crucial role in explaining the immediate effects of scene context on object processing. For instance, Oliva and Torralba^13^ suggested that without the need to identify the objects of a scene, its spatial layout — i.e., “the global shape” of a scene as specified in their Spatial Envelope Model — may quickly convey gist information (à la “seeing the forest before the trees”^14^). Likely, the visual system uses a combination of local (such as objects and their expected co-occurrences) and global scene properties for scene and object recognition (for a review, see^15^). Here, we will focus on the role of global image statistics contained within scenes. To put the influence of global image statistics to an even stronger test, we will assess the influence of visual representations of scenes, so-called textures of scenes, which retain some statistical features of the scene while discarding spatial layout information, as outlined in the following paragraph.

**Figure 1:**
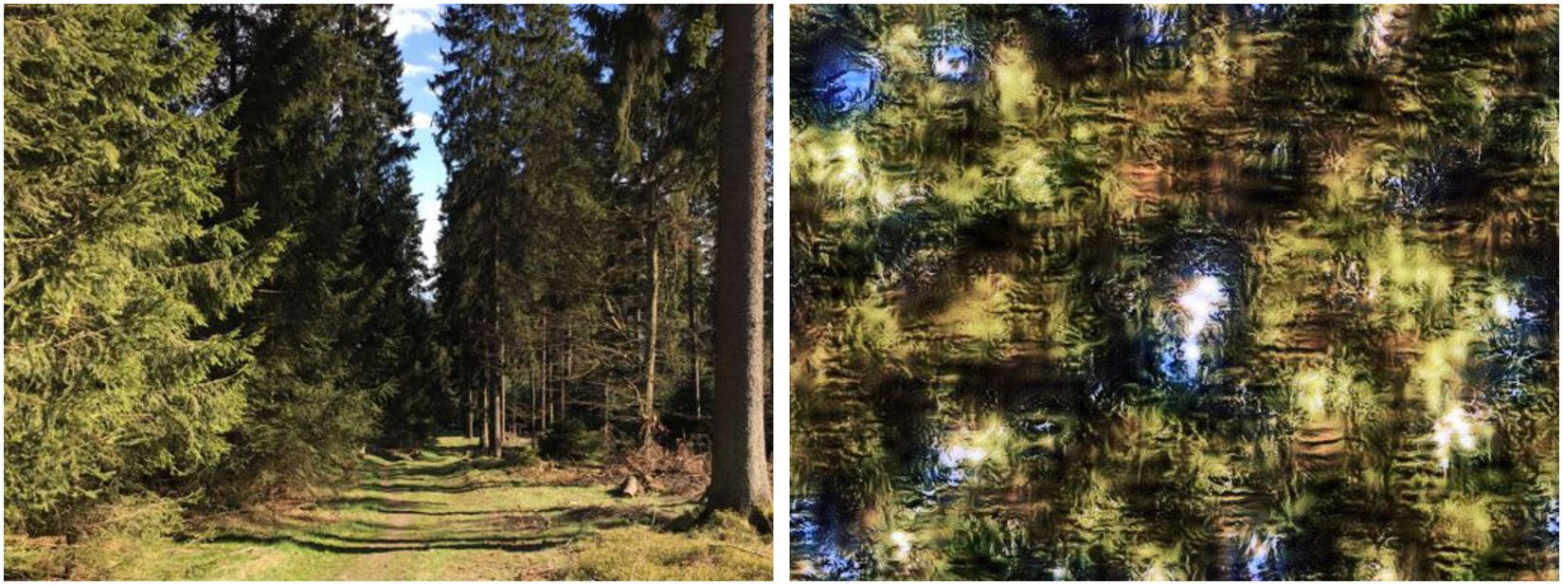
Example of a texture (right) that preserves similar summary statistics as compared to a forest scene (left) but discards global shape information.

Work on so-called ensemble statistics has shown that within a glance we can extract statistical information from a set of items^16^. For example, the visual system accurately estimates the average size and orientation of a set of objects without attending to each of them individually^17^. Apart from this item-based information, which requires prior segmentation of the display, one can also extract a “summary” of global information from a whole scene, without segmentation. One may extract global properties from scenes in a feedforward manner, for example the degree of naturalness, as has been shown in behavioural experiments^18,19^ and further supported by computational modeling work^13^. A recent study showed that global ensemble textures, that is, representations of multiple localized spatial frequency and orientation distributions, are sufficient to activate scene representations and influence object processing behaviourally^20^. Whereas this study investigated the influence of global image properties while retaining spatial layout information, we used textures as introduced by Portilla and Simoncelli^21^ which retain a collection of global statistical measures — based on basic visual features — similar to a source image, but do not convey global shape information (see Fig. 1).

Portilla and Simoncelli^21^ developed a parametric model (hereafter referred to as the Portilla and Simoncelli model or P-S model) to characterize and reconstruct the appearance of visual textures (i.e., homogenous images that contain recurrent elements such as a stone wall). The P-S model extracts four sets of statistics from an input image (here together called summary statistics) that are used to create a synthesized texture version of it: *First-order statistics* capture the luminance and spectral information of the original image. *Second-order statistics* are based on the local autocorrelation and convey periodically repeating image structure. *Magnitude correlation* measures co-occurring image structure across position, orientation, and scale. Finally, *phase statistics* are computed across scales to capture a more thorough three dimensional appearance (for a more detailed description of the parameters, see^21^). These sets of statistics are then iteratively imposed on Gaussian noise until the generated texture has similar statistical properties as the input image. Importantly, the P-S model keeps only the local structure of the input image (e.g., edges and periodic patterns), while its global 3D configuration, if any, is lost^22^.

Could such visualizations of summary statistics as yielded by the P-S model be enough to influence semantic scene and possibly even object processing? While the P-S algorithm was originally not developed to study scene understanding, it turns out to be an excellent tool for investigating whether summary statistics are sufficient for influencing semantic scene and possibly even object processing: Naturalistic scenes are not homogenous and therefore the algorithm discards the spatial relations of a scene’s basic visual features and ultimately its global shape^23^, rendering it apparently meaningless^24^, while selectively preserving the basic visual features on the local level.

**Figure 2:**
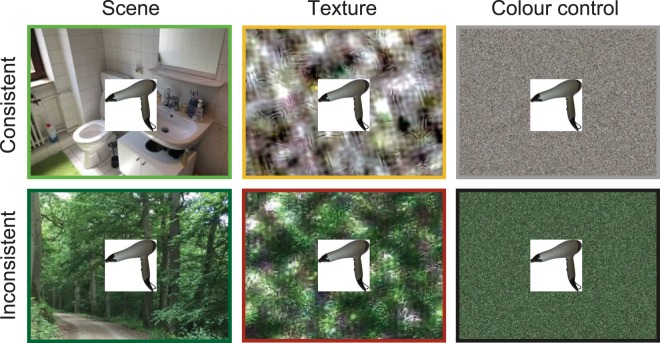
Examples of consistent and inconsistent real-world scenes, textures, and colour controls.

Here we conducted three experiments to investigate whether seeing a scene’s global summary statistics — without any spatial layout information — is sufficient for explicit scene categorization at the superordinate and basic levels, and whether it influences object identification. In Experiment 1, participants were presented with real-world scenes, their texture versions, or colour controls (i.e., scrambled scenes lacking the higher order statistics of the original scenes), and explicitly categorized the images at the superordinate level (as indoor vs. outdoor) and at the basic level (as one of eight scene categories: kitchen, bathroom, bedroom, office, forest, mountain, beach or street). If summary statistics contain some scene semantic information, we should see increased categorization performance for the texture compared to the control condition — at least at the superordinate level. In Experiment 2, a rapid object recognition experiment, participants were asked to name thumbnail images of objects, which were briefly presented on top of consistent or inconsistent real-world scenes, textures, or colour controls (see Fig. 2). Moreover, participants indicated how confident they were about their responses. If textures provide sufficient scene information to affect object identification, we should see diminished naming performance and confidence ratings not only for inconsistent (vs. consistent) scenes, but also inconsistent textures, while no such consistency effect should be observed for the control condition. In Experiment 3, we recorded ERPs from observers presented with the same stimuli as in Experiment 2 to test whether presenting isolated object thumbnails superimposed on inconsistent background scenes would yield an increased N300/N400 response similar to the ones reported previously^6–9^. More importantly, we wanted to investigate whether objects on inconsistent textures also trigger an N300/N400 response, which would imply object processing difficulties due to background information that does not carry obvious semantic meaning.

## Experiment 1

In Experiment 1, we tested how well textures containing the mere summary statistics of scenes can be categorized compared to both original scenes and colour controls.

### Results and Discussion

**Figure 3:**
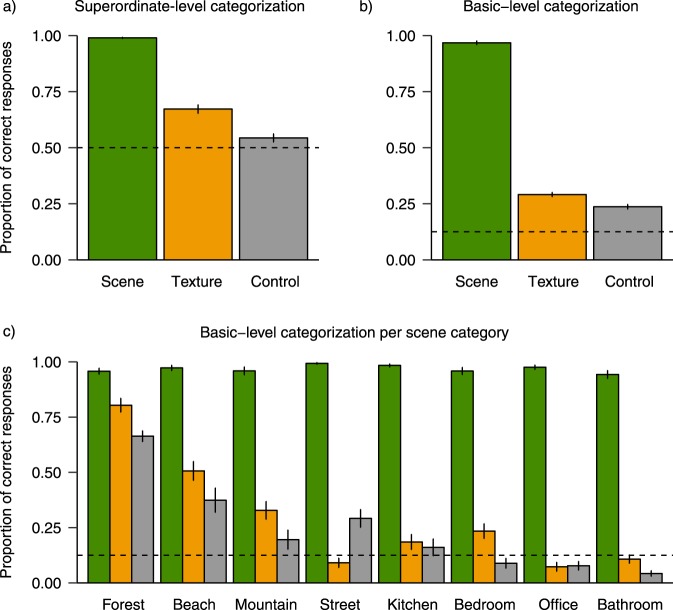
Proportion of correctly categorized scenes, textures, and colour controls for superordinate categorization (**a**) basic-level categorization (**b**) and basic-level categorization per scene category (**c**). Error bars depict the standard error of the mean. Dashed lines indicate chance levels.

As dependent measures, we calculated the proportion of correct responses at the superordinate (indoor vs. outdoor) and basic level (forest, beach, etc.) categorizations (see Fig. 3a,b). For each dependent measure, binary single trial responses (correct/incorrect) per participant were submitted to a generalized linear mixed-effects model (GLMM) (details about model selection can be found in the Data Analysis section). At the superordinate level, textures were categorized more accurately than colour controls, *ß* = −0.764, SE = 0.132, *z* = 5.798, *p* < 0.001. At the basic level, we did not find a significant difference between textures and colour controls, *ß* = −0.396, SE = 0.291, *z* = 1.361, *p* = 0.174. Categorization performance for real-world scenes was at ceiling, and this lack of variance led to degenerate models in a multilevel model approach. Thus, comparisons with the scene condition were submitted to two-sided paired t-tests: At the superordinate level, scenes were categorized better than both textures, *t*(23) = 17.38, *p* < 0.001, and colour controls, *t*(23) = 27.3, *p* < 0.001. At the basic level, scenes were also categorized better than textures, *t*(23) = 57.64, *p* < 0.001, and colour controls, *t*(23) = 60.4, *p* < 0.001.

Comparisons against chance levels revealed the following: At the superordinate level, scenes, *t*(23) = 209.4, *p* < 0.001, textures, *t*(23) = 9.52, *p* < 0.001, but not colour controls, *t*(23) = 2.6, *p* = 0.016, were categorized above chance (assuming a chance level of *µ = *0.5). At the basic level, scenes, *t*(23) = 108.02, *p* < 0.001, textures, *t*(23) = 17.33, *p* < 0.001, and colour controls, *t*(23) = 11.89, *p < *0.001, were categorized above chance (assuming a chance level of *µ = *0.125).

These results suggest that scene summary statistics were used during explicit categorization of texture images, at least at the superordinate level: Indoor vs. outdoor performance was significantly increased in the texture condition compared to the colour control condition. Summary statistics therefore seem to convey at least some scene semantic information that is not present in colour control images, which lack the higher order statistics of the original scenes. At the basic level (Fig. 3b), however, we did not find such an effect: Textures were only numerically categorized better than colour controls. Figure 3c depicts categorization performance as a function of scene category. Overall, outdoor images, especially forests and beaches, indicate an advantage in categorization performance over indoor scenes, even for colour controls. Reviewing the stimulus material, forests and beaches indeed seem to be the two categories with the most distinct colours. We therefore deem it possible that the overall above-chance performance for both textures and controls (as depicted in Fig. 3b) is mainly driven by the diagnostic colours of forests and beaches. From inspecting within-category performance in Fig. 3c, it becomes apparent that summary statistics, as preserved in textures, may have been exploited more for some categories than for others. These findings for the basic-level categorization performance should be interpreted with caution due to the sequential procedure: Participants always performed the superordinate categorization first, which might have influenced the basic-level decision.

As perceiving mere scene summary statistics — which seem to lack obvious scene semantics — yielded increased explicit categorization performance compared to colour controls at the superordinate level, we asked whether the presence of summary statistics might be sufficiently strong to also influence the processing of objects superimposed on such textures. Therefore, we conducted a rapid object recognition experiment using the same backgrounds to clarify the role of scene summary statistics in object processing.

## Experiment 2

In Experiment 2, we examined if mere summary statistics of scenes, as preserved in the textures from Experiment 1, are sufficient to modulate object processing similar to normal scenes, while controlling for colour information.

### Results and Discussion

**Figure 4:**
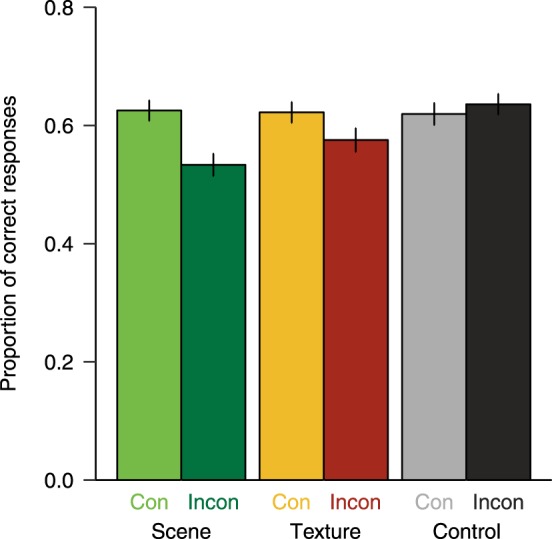
Proportion of correctly named consistent (Con) and inconsistent (Incon) objects which were superimposed on scenes, textures, and colour controls. Error bars depict the standard error of the mean.

As shown in Fig. 4, participants named consistent thumbnail objects superimposed on real-world scenes better than inconsistent objects, whereas there was only a slight difference for textures and no effect for colour controls. Binary single trial responses (correct/incorrect) per participant were submitted to a GLMM. On scenes, consistent objects were named more accurately than inconsistent objects, *ß* = −0.550, SE = 0.279, *z* = −1.969, *p* = 0.049. We did not find a significant consistency effect for textures, *ß* = −0.169, SE = 0.279, *z* = −0.605, *p* = 0.545, and colour controls, *ß* = 0.007, SE = 0.280, *z* = 0.026, *p* = 0.979.

Confidence ratings for count data ranging from one (very unconfident) to six (very confident) were submitted to a GLMM. In the scene condition, we found numerically higher confidence ratings for responses to consistent objects relative to inconsistent objects but the effect was not statistically significant, *ß* = −0.102, SE = 0.058, *z* = −1.756, *p* = 0.079. Textures did not show a significant consistency effect on confidence ratings either, *ß* = −0.037, SE = 0.058, *z* = −0.632, *p* = 0.527, and neither did the control condition, *ß* = 0.016, SE = 0.058, *z* = 0.277, *p* = 0.782.

The finding that object naming performance is better for objects on consistent scenes replicates previous reports of semantic consistency effects^2,3^. Contrary to previous reports, however, the current procedure involved thumbnails of isolated objects superimposed on scenes rather than objects embedded in scenes. Furthermore, our results suggest that neither similar summary statistics, as preserved in the textures, nor colour information (control background) helped or hindered object recognition. However, despite the lack of a statistically significant difference in the GLMM, inconsistent textures yielded less correct object identifications numerically. While object naming accuracy for consistent objects was quite similar across the different types of background images, a decrease in performance is visible for inconsistent objects on scenes and textures. It seems that the impairment of object recognition (as opposed to facilitation) drives the consistency effect in the current paradigm. Possibly, our behavioural methods were not sensitive enough to capture consistency effects for textures. While the mere summary statistics of inconsistent scenes might not yield significant behavioural effects on object recognition, the semantic processing of objects could still be affected on the neuronal level. Therefore, we conducted an ERP experiment to test whether a neurophysiological measure could shed more light on the nature and temporal progression of semantic object processing as a function of scene context information. Specifically, we aimed to look at the N400 component – a sensitive marker for impaired semantic object processing.

## Experiment 3

In Experiment 3, we examined whether inconsistent vs. consistent objects on textures (which contain similar summary statistics as non-texturized scenes but only little semantic content) elicit similar N300/N400 responses as typically seen for inconsistent objects within scenes^6–9,11^. We used a Repetition Detection Task^9^ (see Procedure, Experiment 3) which is well-suited for ERP experiments, allowing for a late stimulus offset (e.g., 2000 ms object-scene presentation). Moreover, the task keeps participants attending to the stimuli, without making the consistency manipulation task-relevant to them^9^. Furthermore, the task ensured that both objects and backgrounds were attended, unlike in Experiment 2 where background images were task-irrelevant.

### Results and Discussion

Single trial analysis was restricted to the mid-central region (averaged over the electrodes FC1, FC2, Cz, CP1, CP2) as it has previously been associated with pronounced N400 responses^6,9^. A mean amplitude per participant, per trial was calculated for the N300 (250–350 ms) and N400 (350–600 ms) time windows^7–9^. A separate linear mixed-effects model (LMM) for the means of each of the two time windows was conducted.

#### Behavioural results

In total, the Repetition Detection Task yielded 70% hits (*i.e*., exact repetitions were detected) and a false alarm rate of 12%.

#### EEG results

In the N300 time window (250–350 ms), inconsistent objects on real-world scenes elicited a significantly more negative potential than consistent objects, *ß* = −1.538, SE = 0.416, *t* = −3.698, 95% CI [−2.353, −0.723]. For textures, we found a significant difference as well, *ß* = −0.822, SE = 0.421, *t* = −1.953, 95% CI [−1.647, −0.003], whereas colour controls did not show any difference, *t* < 1. In the N400 time window (350–600 ms), inconsistent objects on real-world scenes elicited a significantly more negative potential than consistent objects, *ß* = −1.628, SE = 0.409, *t* = −3.983, 95% CI [−2.428, −0.827]. For textures, we found a significant difference as well, *ß* = −1.305, SE = 0.414, *t* = −3.156, 95% CI [−2.116, −0.495]. Colour controls, in contrast, did not elicit a significant N400 response, *t* < 1.

**Figure 5:**
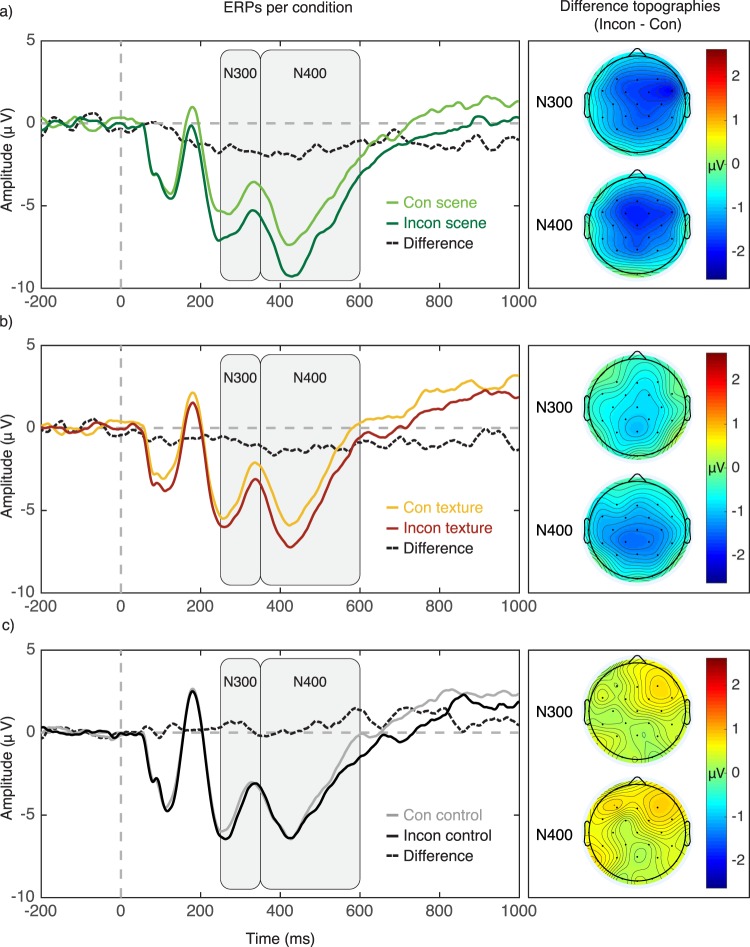
Grand-average event-related brain potentials (ERPs) recorded from the midcentral region (electrodes FC1, FC2, Cz, CP1, CP2) for semantically consistent (Con) vs. inconsistent (Incon) scenes (a, left panel), textures (b, left panel) as well as colour controls (c, left panel), and corresponding difference topographies for the N300/N400 time windows (right panels).

Inconsistent relative to consistent objects evoked a significantly more negative potential in the N300 as well as in the N400 time window. Objects on textures elicited less pronounced N300/N400 responses, whereas the control condition did not show any effect in either time window. Figure 5 shows the grand-average ERPs per condition (left panels) and corresponding averaged scalp topographies of the difference between consistencies per time window (right panels). In the N300 time window, the scene condition reveals a pronounced fronto-central negativity with a right-lateralized maximum for object-scene inconsistencies. The texture condition shows only a minor central/occipito-central negativity and colour controls do not show any negativity. In the N400 time window, the scene condition reveals a pronounced fronto-central negativity, and, for textures, we see a substantial, however less pronounced and slightly more occipital negativity. Colour controls do not show such negative potentials. These results suggest that object processing is affected by the background scene, even for superimposed isolated objects (here surrounded by a white background). More importantly, the mere summary statistics of these scenes seem to be sufficient to influence object processing to some degree as well, as can be seen in a similar, though less pronounced N400 response. Mere colour information (control condition) does not seem to influence object processing.

## General Discussion

Scene context has shown to affect object processing1^1,2,3,6–9,11^ but so far it has remained unclear what role a scene’s low-level features play. We conducted three experiments to shed light on the question whether the mere summary statistics of a scene, lacking obvious semantic meaning and spatial layout information, are sufficient to facilitate explicit scene categorization and modulate the semantic processing of an object in its visual context. To answer this question, in Experiment 1, participants categorized (1) real-world scenes, (2) textures of scenes which contained similar summary statistics, and (3) colour controls which lacked the higher-order statistics of the scenes they were derived from. Participants categorized these images at the superordinate (indoor vs. outdoor) and basic (kitchen vs. bathroom vs. office etc.) level. We found that textures yielded better performance than colour control images at the superordinate level, suggesting that they contain at least some scene semantic information. Note, however, that the above chance performance at the basic level for both textures and controls might have been driven by substantially better categorization performance for a subset of image categories.

In Experiment 2, we asked whether scene summary statistics could influence object recognition. Therefore, we briefly presented participants with objects superimposed on real-world scenes, textures of scenes, and colour controls. We found that, superimposed on scenes, semantically consistent objects were named more accurately than inconsistent objects. Our findings for intact scenes are in line with previous studies^2,3^ and now additionally show that such consistency effects generalize to thumbnail images of isolated objects which are not embedded in a scene (regarding scale, position, orientation, lighting conditions etc.) but are presented superimposed on it. This suggests that even when the scene context is physically separated from the critical object — unlike object cut-outs or actually integrated objects used in previous studies — it may influence semantic object processing as shown by modulated object-naming performance and confidence ratings.

Objects on textures showed non-significant effects in the same direction, whereas objects on colour control backgrounds showed no effects at all. This indicates that taking away most of the semantic information and preserving only a scene’s global summary statistics was insufficient to influence object-naming performance behaviourally and suggests that other sources of scene information are exploited to facilitate object recognition in context. However, it remained unclear whether our behavioural measure was sensitive enough to capture possibly subtler consistency effects for textures. Unlike in Experiment 1, stimuli were only briefly presented (56 ms) in Experiment 2, and the tasks differed (explicit forced choice scene categorization vs. naming objects). Moreover, the object-naming task in Experiment 2 rendered the background scene task-irrelevant (and participants were informed that it was irrelevant), making it less likely to affect performance.

In Experiment 3, we therefore further investigated whether texturized scenes contain sufficient scene information to affect object processing using EEG as a possibly more sensitive, online measure of object- and scene semantic processing. We specifically looked at the N400 ERP component, since it has previously been used to investigate semantic processing of both words in sentences^4,5^ as well as objects in scenes^6–9^ (for a review see^10^). Moreover, in Experiment 3, participants had to attend to both the object and the background image in order to complete a Repetition Detection Task^9^. When objects were presented on real-world scenes, we found a pronounced N400 response for inconsistent relative to consistent objects in the mid-central brain region. This implies that the background scene modulated the semantic processing of the foreground object. Contrary to the lack of a behavioural effect in Experiment 2, we did find an N400 effect for inconsistent objects presented on texturized scenes, whereas we found no evidence for such an effect for colour controls.

Based on the similar time-course of the N400 response to intact scenes compared to texturized scenes, one might take this as evidence for similar mechanisms underlying object processing for both types of background images. During the earliest stages of processing, participants may have been able to identify the gist of the scene, at least to the level of indoor vs. outdoor discrimination^18^. Subsequently, the gist of the scene may have quickly activated scene knowledge that in turn provided top-down predictions regarding semantically related objects^15,25^. The presentation of an unlikely object might have then triggered semantic integration difficulties as seen in the increased N400 responses for both scene and texture backgrounds.

Considering that the P-S model discards much information (e.g., the global shape) and given the drastically reduced categorization performance for texturized scenes in Experiment 1 (−32%) compared to scenes at the superordinate level), it seems somewhat surprising that these less meaningful textures still yielded an N400 response that was comparable to the one seen for intact, meaningful scenes. The fact that the N400 in response to inconsistent objects on textures followed a similar time-course but was just less pronounced could be taken as an indication that the magnitude of the N400 effect is influenced by the amount and/or the type of scene information conveyed by the background. As shown in Experiment 1, textures of scenes may carry some scene semantic meaning, such as information regarding the naturalness of scenes^18^, or in some cases even the exact category (e.g., for forests and beaches). That type of meaning might in turn provide predictions regarding semantically related objects, at least on a crude level (e.g., seeing outdoor scenes triggers predictions about any outdoor objects).

In addition to the modulated N400 response, an N300 effect was present for scenes as well as for textures. The N300 has previously been suggested to reflect context effects on object processing at a rather perceptual level^11^ resulting in impeded identification of semantically inconsistent objects. While the exact N300/N400 distinction is debatable^12^, this notion would imply that both scenes and textures yielded pre-semantic processing difficulties. Once identified, processing an object that does not match semantic predictions elicited by the scene context (e.g., the item on the kitchen counter turns out to be a roll of toilet paper), may have impeded the semantic integration of this particular object resulting in increased integration costs as seen in the increased N400 response.

Note that in the analyses of Experiment 3, we only compared indoor scenes to outdoor scenes. The images in these categories have clearly different statistical properties. Not only do indoor scenes contain many more objects than outdoor scenes^26^ but their second-order statistics also differ^27^. Man-made scenes, for instance, contain more energy at high spatial frequencies than natural scenes as sharp edges occur more frequently. Moreover, second-order statistics vary as a function of scene scale. If the scale is large, like in many outdoor scenes, the image likely has an increased amount of energy at low spatial frequencies (note, that the horizon might occupy a large proportion of the image and objects may appear less sharp). It would be interesting to see whether the reported EEG effects are similar for comparisons between basic-level scene categories. Unfortunately, the number of trials per scene category in Experiment 3 did not allow for an inspection of ERP responses to individual scene categories, such as forests and beaches. Finally, in order to test whether we would be able to replicate the elicitation of the N400 component by semantically inconsistent objects using thumbnails of isolated objects on white backgrounds superimposed on background scenes, we decided to make both the background and the objects task-relevant. It would be interesting to see whether we would still be able to find effects in ERPs for mere textures of scenes when the scene background is rendered irrelevant by the task.

In addition, future experiments could look at basic-level scene categories individually, and more subtle inconsistencies could be investigated (e.g., within indoor-inconsistencies such as a printer in an office vs. a printer in a kitchen). Importantly, specifying the differential role of other sources of information — such as the global shape of a scene or the influence of other co-occurring objects — could yield further insights into the nature and progression of semantic identification and integration of objects and scenes.

## Conclusion

Together, the experiments presented here suggest that the presence of mere summary statistics of a scene might be sufficient to extract at least some scene semantic information, which in turn can aid above chance scene categorization at the superordinate level and might also contribute to the previously reported semantic consistency effects found in object recognition tasks. Using ERPs we found further evidence that the summary statistics of a scene can be sufficient to affect semantic object processing: Objects presented on semantically inconsistent textures of scenes, which lack obvious meaning, triggered an N400 response that is less pronounced, but otherwise similar to the N400 repeatedly reported for unmodified scenes. These results suggest that the summary statistics of a scene could be one of the contributing ingredients of a scene — besides spatial layout, co-occurring object information etc. — that modulate the semantic processing of objects therein.

## General Method

### Participants

In each experiment, we analysed data from 24 participants (Experiment 1: mean age = 21.75 years, *min* = 18, *max* = 26, 20 females; Experiment 2: mean age = 22.02 years, *min* = 18, *max* = 34, 10 females; Experiment 3: mean age = 21.17 years, min = 18, max = 33, 20 females). One additional participant who did not follow instructions was excluded from Experiment 1. Another one was excluded from Experiment 2 because of major difficulties doing the task, resulting in low performance. Eight additional participants were excluded from Experiment 3 as they scored poorly in the Repetition Detection Task, did not follow instructions, or had noisy EEG signals. Participants who took part in Experiment 1 were paid volunteers or participated for partial course credit. All participants were German native speakers, had normal or corrected to normal vision (at least 20/25 acuity) and normal colour vision. Participants were naïve to the purpose of the study, unfamiliar with the images that served as stimuli, and gave informed consent prior to participating. All aspects of the data collection and analysis for all three experiments reported here were carried out in accordance with guidelines approved by the Human Research Ethics Committee of the Goethe University.

### Stimulus material and design

We collected 252 real-world scenes (1024 × 768 pixels) from different indoor and outdoor categories on the internet (using Google search and the LabelMe database^28^). While the number of indoor and outdoor scenes was equal, the number of scenes per basic-level category was not perfectly balanced (33 kitchens, 33 bathrooms, 27 bedrooms, 33 offices, 29 forests, 34 mountains, 30 beaches, 33 streets) as a result of a pilot image rating experiment in which only scenes that were sufficiently consistent or inconsistent with respect to the objects used in Experiments 2 and 3 were selected.

Additionally, we generated a synthesized version of each scene — a texture preserving similar global summary statistics but no spatial layout information — by applying the colour version of the P-S algorithm (one large pooling region over the whole image)^21^. A colour control condition, which preserved the same colours as the original scene but lacked its higher-order statistics (i.e., magnitude- and phase correlation), was created by randomly shuffling all pixels within the image in blocks of 2 × 2 pixels.

For Experiment 1, scenes, textures, and colour controls were randomly assigned to participants using a 3-by-1 Latin square design. A grey square of the size 256 × 256 pixels was inserted in the centre of each image to keep the visual information similar to Experiments 2 and 3, where an object would appear in that spot.

In Experiments 2 and 3, we assigned a semantically consistent 256 × 256 pixel thumbnail of an isolated object on a white background^29–32^ to each scene. To create semantic inconsistencies, we paired indoor and outdoor scenes such that the same object was consistent with one scene, but inconsistent with the other (see Fig. 2). Objects were superimposed on the scenes rather than embedded in them to control for potential variation in figure-ground segmentation of the objects in respect to the different types of background images. All scenes were randomly assigned to the six conditions (consistent scene, inconsistent scene, consistent texture, inconsistent texture, consistent colour control, inconsistent colour control) and counter-balanced across participants using a 3-by-2 Latin square design. For Experiment 2, four masks (1024 × 768 pixels each) that contained random squares were generated^33^.

### Apparatus

In Experiment 1, where timing was less crucial, stimuli were displayed on a 22-in. monitor with a resolution of 1680 × 1050 pixels and a refresh rate of 60 Hz. In Experiments 2 and 3, stimuli were displayed on a 24-in. monitor with a resolution of 1920 × 1080 pixels and a refresh rate of 144 Hz. Presentation was controlled by MATLAB, making use of the Psychophysics Toolbox^34,35^. Viewing distance was 60 cm in all three experiments. In Experiment 1, scenes spanned an angular size of 25.8° horizontally and 19.57° vertically. In Experiments 2 and 3, scenes spanned an angular size of 26.56° horizontally and 20.03° vertically, while thumbnail objects spanned 6.75° horizontally and vertically.

### Data Analysis

Behavioural and EEG single trial data were analysed with generalized/linear mixed-effects models using lme4^36^, a package provided for the statistical computing software R^37^. In Experiment 1, we applied a GLMM with a Binomial distribution, modelling three planned contrasts per categorization level (superordinate-level, basic-level): scenes vs. textures, scenes vs. controls, and textures vs. controls. In Experiment 2, we applied a GLMM with a Binomial distribution for object naming accuracy and a GLMM with a Poisson distribution for confidence ratings, each modelling three difference contrasts: consistent objects were compared against inconsistent objects per background type (scenes, textures, colour controls). P-values for GLMMs were obtained from asymptotic Wald tests. In Experiment 3, we applied a LMM, modelling three planned difference contrasts: consistent objects were compared against inconsistent objects per background type. Confidence intervals were computed with the *confint* function in R.

We chose the mixed models approach as it allows between-subject and between-item variance to be estimated simultaneously and thus yields advantages over traditional F1\ F2 analysis of variance^38,39^. In our case, it offers the possibility to incorporate participants, scene categories, and items as random effects in the model. Maximum likelihood estimation was used to fit all models. Planned sum contrasts (Experiment 1) and difference contrasts (Experiments 2 and 3) were defined to analyse the comparisons of interest (see Results sections). In the case of sum contrasts, the intercept was defined as the grand mean of the respective dependent measure; slope coefficients depict the difference between factor levels. As random effects, we included participants, scene categories, and items (i.e., individual scenes). The random effects structure was initially chosen to be maximal^40^: We included random intercepts for participants, scene categories and items, as well as random slopes for participants and scene categories. In practice, models with random intercepts and slopes for all fixed effects often fail to converge or lead to overparameterization^41^. In order to produce models that converge on a stable solution and are properly supported by the data, we used a Principal Components Analysis (PCA) of the random-effects variance-covariance estimates for each fitted mixed-effects model to identify overparameterization^41^. Random slopes not supported by the PCA and not contributing significantly to the goodness of fit (likelihood ratio tests), were removed from the model. The best fitting model’s retained variance components per experiment were as follows: In Experiment 1, at the superordinate level, participants, scene categories and items intercepts were included, as well as by-participant random slopes for background type (texture, control). At the basic level, participants, scene categories and items intercepts were included, as well as by-scene category random slopes for background type (texture, control). In Experiment 2, participants, scene categories and items intercepts were modelled, as well as by-scene category random slopes for consistency (consistent, inconsistent). In Experiment 3, participants, scene categories and items intercepts were considered, as well as by-participant random slopes for background type (scene, texture, control). One-sample t-tests against chance levels (Experiment 1) were Bonferroni corrected with an alpha level of 0.05/6 = 0.0083.

## Experiment 1

### Procedure

**Figure 6:**
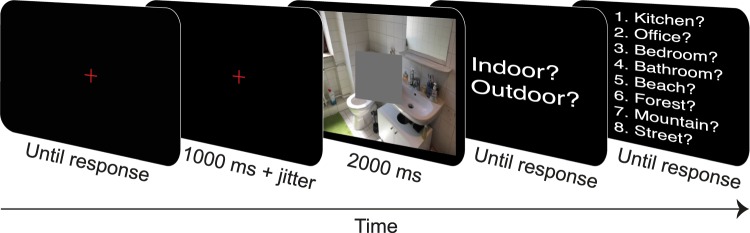
Trial sequence of the categorization experiment (slightly adapted for display purposes).

Participants were instructed that they would see a set of images comprising photographs as well as artificial images generated from photographs, and completed 252 experimental trials (see trial sequence in Fig. 6). On each trial, a fixation cross was presented. Upon keypress the fixation cross remained for 1000–1300 ms. Subsequently the image (either scene, texture, or colour control) was presented for 2000 ms. Participants had been instructed to fixate the central grey square in this period. Then, they were asked to categorize the image at the superordinate level, followed by basic-level categorization, both by keypress.

## Experiment 2

### Procedure

**Figure 7:**
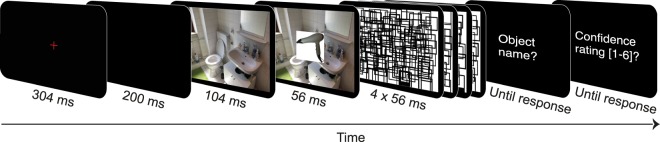
Trial sequence of the rapid object recognition task in Experiment 1 (slightly adapted for display purposes).

All participants completed six practice and 252 experimental trials (see Fig. 7). In the beginning of each trial, a fixation cross was presented for 304 ms, followed by a blank screen for 200 ms, a preview of the background image (either a scene, texture or colour control) for 104 ms, the critical object (either consistent or inconsistent) on the same background image for 56 ms, immediately followed by four random masks for 56 ms each. Then an input panel appeared, prompting participants to type in the object’s name. Participants were instructed to name the objects as accurately and precisely as possible (e.g., “cupboard” instead of “furniture”), using only a single German word. It was emphasized that background images were task-irrelevant. To proceed, the object name needed to be validated by pressing the return key. Participants were instructed to guess if they were uncertain about an object or had missed it. Subsequently they were asked how confident they were about their response on a scale from one (very unconfident) to six (very confident). The next trial started upon keypress. Participants were instructed not to move their eyes from the centre of the screen during the preview as well as during the object and background presentation.

### Preprocessing

First, three independent raters who were unaware of the experimental conditions evaluated the responses. Raters were instructed to consider responses as correct if they matched a sample solution (generated in a preliminary study where five experimenters had agreed upon a correct name for each object) or a synonym of it, regardless of spelling mistakes. Less precise responses (e.g., “fruit” instead of “apple”) were supposed to be considered as incorrect. If the majority of the raters considered a response as correct it was deemed correct; otherwise it was incorrect.

## Experiment 3

### EEG setup

We used 32 active electrodes (actiChamp, Brain Products) to record the electroencephalogram (EEG) from the scalp. Electrodes were positioned according to the 10–20 system. Two electrodes placed on the left and right mastoids served as reference and one electrode below the left eye served as an EOG channel. All signals were recorded at a sampling rate of 1000 Hz.

### Procedure

**Figure 8:**
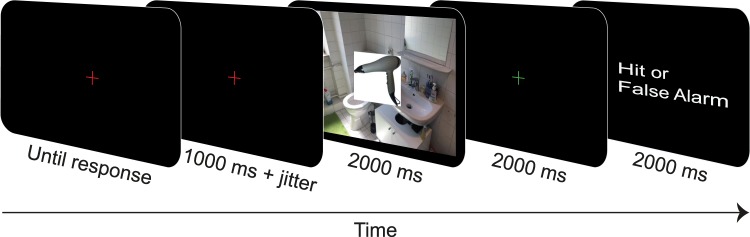
Trial sequence of the ERP experiment (slightly adapted for display purposes).

Participants were told that they would see a set of images comprising photographs as well as artificial images generated from photographs, and that superimposed on each of these images an isolated object would be presented in the centre. In the beginning of each trial, a red fixation cross appeared on the screen, indicating that blinking was encouraged at this point. Upon keypress, the fixation cross remained on the screen for another 1000–1300 ms. Then, the background image (either scene, texture, or colour control) and the critical object (either consistent or inconsistent) were presented simultaneously for 2000 ms (see Fig. 8). Participants had been instructed not to move their eyes from the centre of the screen and not to blink during this time.

Then, while a green fixation cross was presented for 2000 ms (immediately after the object-background presentation), participants were instructed to press a key if they had seen an exact repetition (i.e., a certain object appearing for the second time on a certain background image) but not when they spotted a novel combination of scene and object or a lure (either the same object presented again but on a different background image or the same background presented again but paired with a different object). If the key was pressed, participants received feedback on whether the response was a hit or a false alarm. All backgrounds and objects that were included in the Repetition Detection Task (16 exact repetitions, 8 lures) were collected in addition to the main stimulus set.

### Preprocessing

Bad channels, as judged by visual inspection, were interpolated (this never amounted to more than five electrodes per participant). EEG signals were band-pass filtered at 0.1–112.5 Hz and notch filtered at 50 Hz offline. 1200 ms epochs were then extracted, time locked to stimulus onset (−200 ms to 1000 ms) and baseline corrected by subtracting the mean voltage in the 200 ms prior to stimulus onset. Epochs that were part of the Repetition Detection Task or were falsely reported as repetitions (M = 1.17%, min = 0%, max = 3.97% of experimental epochs) were excluded from analysis. Moreover, experimental epochs containing artefacts such as eye blinks or eye movements were rejected with a semi-automatic procedure^42,43^: Firstly, epochs in which the signal exceeded a certain threshold, which was tailored to each participant’s data (see Supplementary Table S1), at any channel, were removed. Secondly, epochs in which the maximum peak-to-peak voltage in a moving window of 200 ms, which slid across the epochs in steps of 50 ms, exceeded a certain treshold (Supplementary Table S1) at any channel were removed. In total, 9.32% of all included epochs were rejected (min = 1%, max = 20%; the percentage of rejections was not significantly different across conditions, *F*(3.59, 82.57) = 1.27, *p = *0.289; Greenhouse-Geisser correction was applied). Remaining epochs were submitted to a LMM. For display purposes, the data was aggregated into an average waveform per participant, per condition, low-pass filtered at 30 Hz, and subsequently grand-averaged per condition.

## Electronic supplementary material

Supplementary Information

## Data Availability

The data analysed in the current study are available for download: https://osf.io/5wxkg/?view_only=8019580396714f79ab8f3ad461416c43.
