# Supplementary material for: The role of scene summary statistics in object recognition
Source: Sci Rep. 2018 Oct 2;8:14666. doi: 10.1038/s41598-018-32991-1 (PMC6168578; doi:10.1038/s41598-018-32991-1)
Supplement: Supplementary file 1 — Supplementary Information [file 41598_2018_32991_MOESM1_ESM.doc]

**Supplementary Information**The role of scene summary statistics in object recognition

Tim Lauer, Tim H. W. Cornelissen, Dejan Draschkow, Verena Willenbockel
& Melissa L.-H. Võ

**Supplementary Table S1**

*Artifact rejection thresholds per participant*

| Participant | Abosolute threshold | | | Moving window |
| --- | --- | --- | --- | --- |
| Number | Lower bound | Upper bound | Treshold | |
| 1 | -100 | 100 | 100 | |
| 2 | -100 | 100 | 70 | |
| 3 | -100 | 100 | 80 | |
| 4 | -100 | 100 | 100 | |
| 5 | -100 | 100 | 100 | |
| 6 | -100 | 100 | 100 | |
| 7 | -100 | 100 | 80 | |
| 8 | -100 | 100 | 100 | |
| 9 | -100 | 100 | 100 | |
| 10 | -100 | 100 | 70 | |
| 11 | -100 | 100 | 100 | |
| 12 | -100 | 100 | 100 | |
| 13 | -100 | 100 | 80 | |
| 14 | -100 | 100 | 110 | |
| 15 | -100 | 100 | 90 | |
| 16 | -80 | 80 | 100 | |
| 17 | -100 | 100 | 80 | |
| 18 | -100 | 100 | 90 | |
| 19 | -100 | 100 | 100 | |
| 20 | -100 | 100 | 100 | |
| 21 | -100 | 100 | 100 | |
| 22 | -100 | 100 | 100 | |
| 23 | -100 | 100 | 80 | |
| 24 | -100 | 100 | 100 | |

*Note.* Artifacts rejection thesholds were tailored to each participant’s
data to detect as many true artifacts as possible while detecting as few
epochs without true artifacts as possible.
